# Salidroside regulates tumor microenvironment of non-small cell lung cancer via Hsp70/Stub1/Foxp3 pathway in Tregs

**DOI:** 10.1186/s12885-023-11036-5

**Published:** 2023-08-01

**Authors:** Zexin Wen, Tong Liu, Yanli Zhang, Qiujuan Yue, Hang Meng, Yijie He, Yi Yang, Minghao Li, Jianwen Zheng, Wei Lin

**Affiliations:** 1 Department of Medicine, Xizang Minzu University, Xianyang, Shaanxi China; 2 Basic Medical College, Xinjiang Medical University, Urumqi, China; 3 Department of Oncology, The First Affiliated Hospital of Shandong First Medical University, Shandong Provincial Qianfoshan Hospital, Jinan, China; 4 School of Clinical and Basic Medicine, Shandong First Medical University &Shandong Academy of Medical Sciences, Jinan, China; 5 Shandong Key Laboratory of Rheumatic Disease and Translational Medicine, Shandong Lung Cancer Institute, the First Affiliated Hospital of Shandong First Medical University, Jinan, China; 6 Department of Critical-care Medicine, Shandong Provincial Hospital Affiliated to Shandong First Medical University, Jinan, China

**Keywords:** Salidroside, Non-small cell lung cancer, Tumor microenvironment, Treg, Stub1

## Abstract

**Background:**

The treatment of non-small cell lung cancer (NSCLC) is challenging due to immune tolerance and evasion. Salidroside (SAL) is an extract in traditional Chinese medicine and has a potential antitumor effect. However, the mechanism of SAL in regulating the immunological microenvironment of NSCLC is yet to be clarified.

**Methods:**

The mouse model with Lewis lung cancer cell line (3LL) in C57BL/6 mice was established. And then, the percentage of tumor-infiltrating T cell subsets including Treg was detected in tumor-bearing mice with or without SAL treatment. In vitro, the effect of SAL on the expression of IL-10, Foxp3 and Stub1 and the function of Treg were detected by flow cytometry. Network pharmacology prediction and molecular docking software were used to predict the target of SAL and intermolecular interaction. Furthermore, the effect of SAL on the expression of Hsp70 and the co-localization of Stub1-Foxp3 in Treg was confirmed by flow cytometry and confocal laser microscopy. Finally, Hsp70 inhibitor was used to verify the above molecular expression.

**Results:**

We discovered that SAL treatment inhibits the growth of tumor cells by decreasing the percentage of tumor-infiltrated CD4^+^Foxp3^+^T cells. SAL treatment downregulates the expression of Foxp3 in Tregs, but increases the expression of Stub1, an E3 ubiquitination ligase upstream of Foxp3, and the expression of Hsp70. Inhibiting the expression of Hsp70 reverses the inhibition of SAL on Foxp3 and disrupts the colocalization of Stub1 and Foxp3 in the nucleus of Tregs.

**Conclusions:**

SAL inhibits tumor growth by regulating the Hsp70/stub1/Foxp3 pathway in Treg to suppress the function of Treg. It is a new mechanism of SAL for antitumor therapy.

**Supplementary Information:**

The online version contains supplementary material available at 10.1186/s12885-023-11036-5.

## Background

Non-small cell lung cancer (NSCLC) accounts for approximately 85% of all lung cancer cases [1] and is the leading cause of cancer-related deaths in humans [2, 3]. Despite several advances in the treatment of NSCLC, the mortality of lung cancer remains high [4, 5]. Thus, seeking effective treatment approaches for NSCLC is an urgent requirement.

Salidroside (SAL), a glycoside of tyrosol, is isolated from *Rhodiola rosea*. It has antioxidation, anti-inflammatory effects [6], and anticancer functions [7, 8]. Recent studies have reported that SAL inhibits the proliferation and metastasis of lung cancer [9]. SAL on anticancer decreases proliferation and induces apoptosis in A549 cells through its ability to inhibit oxidative stress and p38 [10]. It also suppresses the proliferation and migration of human lung cancer cells through AMPK-dependent NLRP3 inflammasome regulation [11]. Previous studies have shown that SAL regulates the immune response [12]. However, the effect and the mechanism of SAL on the tumor microenvironment of NSCLC are yet to be clarified.

The tumor microenvironment is known to be immunosuppressive [13–15] and includes multiple immunosuppressive factors, such as regulatory T (Treg) cells and some inhibitory cytokines [16–18]. Tregs expressing the X chromosome-linked and linage-specific transcription factor forkhead box protein P3 (Foxp3) are potent immunosuppressive cells and can serve as brakes during immune responses. Several studies on cancer diseases have emphasized that Treg cells recruited in tumor tissues help the cancer cells escape from immunological surveillance. Heterogenetic Tregs with high frequencies in tumor tissues, bone marrow, lymph nodes, or peripheral blood from NSCLC patients is the predictors of disease outcomes [19–21]. Accumulating evidence suggests that suppressing the function of Tregs inhibits the progression of tumors by regulating the tumor micro-environment [22]. Thus, how to regulate immune balance in the tumor microenvironment remains a research hotspot in antitumor therapy.

Foxp3 is a key transcription factor of Tregs. Regulating the expression of Foxp3 inhibits the activation and function of Tregs [23]. The mechanism of how antitumor drugs regulate Tregs’ function is still unclear. Herein, we investigated the antitumor effect of SAL and its effect on the immune microenvironment in mice bearing NSCLC and explored the putative mechanism of SAL on regulating Tregs.

## Methods

### Animals and animal model

Specific pathogen-free C57BL/6 mice (approximately 8–10-weeks-old, with an average weight of 25 g) were obtained from Beijing Vital River Laboratory Animal Technology Co., Ltd (Beijing, China). The mice were acclimatized in our animal facility and maintained under specific pathogen-free barrier conditions. All animal experiments were approved by Animal Care and Use Committee of the First Affiliated Hospital of Shandong First Medical University & Shandong Provincial Qianfoshan Hospital and Shandong First Medical University &Shandong Academy of Medical Sciences (Jinan, China, SYXK20180007) and procedures for animal experiments were carried out in accordance with relevant guidelines and regulations.

On day 0, C57BL/6 mice were inoculated subcutaneously in the right flank with 3LL cells (1 × 10^6^cells/mice) and randomly divided into three groups: phosphate-buffered saline (PBS), paclitaxel treatment (PTX), and treatment (SAL) groups. After 2 weeks, the mice were administered SAL (6 mg/kg·d) or PTX (2 mg/kg·d) by intraperitoneal injection every day to reduce the tumor size and increase the survival of mice bearing 3LL cells (Fig. S1). Primary tumor development was monitored by palpation. The largest perpendicular tumor diameters were measured with calipers at 4 day intervals. The volume of the tumors was calculated (largest diameter×smallest diameter^2^)/2. Then, the animals were sacrificed by cervical dislocation on day 21 or with subcutaneous tumor volumes exceeding 3,000 mm^3^. When the tumors became palpable with a maximum diameter > 3 cm on days 10–12, the mice received subcutaneous injections of SAL, PTX, or PBS at 4 day intervals for 2 weeks. Single-cell suspensions were prepared from the tumor tissues in a single-cell suspension dissociator (DSC-400, RWD Lifescience Co., Ltd, China) for further analysis.

### Cell culture

3LL Lewis lung carcinoma (clone D122) cell line was a kind gift from Professor Chu (Fudan University, Shanghai, China). The cells were cultured in Dulbecco’s Modified Eagle Medium (DMEM) (Gibco BRL, Carlsbad, CA, USA) with 10% fetal bovine serum (Thermo Fisher Scientific Inc., Waltham, MA, USA) at 37 °C in a humidified atmosphere containing 5% CO_2_.

The spleen obtained from female tumor-bearing mice aged 6–8 weeks. The splenic single-cell suspension was prepared First, CD4^+^T cells were purified by LD enrichment column (Redd system), incubated with anti-biotin microbeads, and then collected on Auto MACS (Miltenyi Biotec GmbH, Bergisch Gladbach, Germany). Subsequently, CD4^+^T cells were incubated with labeled anti-CD25 PE for 20 min, washed, and incubated with anti-PE beads for 15 min. The cells were selected on LS column and purified on Auto MACS. The purity of CD4^+^CD25^+^ sorted cells detected by flow cytometry was estimated as 80–90% (Fig. S2A). The purified Treg cells from tumor-bearing mice were expanded with anti-CD3/CD28 beads (Invitrogen, USA) and 500 U/mL IL-2 (PeproTech, USA). Then, the cells were rested with 100 U/mL IL-2 for 2 days and stimulated with the drugs. The effect of drug concentration on Treg activity was determined by flow cytometry to select the appropriate drug concentration (Fig. S2B).

The cells were stimulated by 2.5 µM HSP70-IN-1 [24] (Hsp70 inhibitor) (MedChemExpress, USA) for 32 h, we found that the Hsp70 inhibitor inhibited Hsp70 expression in Treg (Fig. S3). And then, the intervention groups were supplemented with 0.05 mg/mL SAL (Solarbio, China) or 200 ng/mL lipopolysaccharide (LPS) (Sigma, USA).

### Flow cytometry and antibodies

The tumors were weighed, minced into small fragments, and digested in a medium containing 0.1 mg/mL DNase (Sigma-Aldrich) and 1 mg/mL collagenase IV (Sigma-Aldrich) at 37 °C for 1 h [25] to prepare single-cell suspension for analysis by flow cytometry.

Antibodies targeting CD3, CD4, CD8, CD80, CD86, CD25, I-A/I-E, CD11c, and Foxp3 conjugated to the corresponding fluorescent dyes were purchased from eBioscience (San Diego, CA, USA). Single-cell suspensions (1 × 10^6^ cells) were stained with different monoclonal antibodies according to the manufacturer’s instructions. Finally, the samples were analyzed on a FACS Suite using the Cell Quest data acquisition and analysis software (BD Biosciences, CA, USA).

### Network pharmacology and molecular docking analyses

The chemical constituents and potential targets of SAL were obtained from traditional Chinese medicine database (HERB) (http://herb.ac.cn/). DisGeNET (https://www.disgenet.org/) and GeneCards (https://www.genecards.org/) databases were used to determine the target genes in NSCLC. And then the network topology between potential targets of SAL and the target genes in NSCLC is analyzed by using STRING database (http://string-db.org/) to select the target of SAL acting on NSCLC. The Metascape bioinformatics database was used to analyze the GO molecular function. Finally, PyMOL software was used to predict the binding ability of drug ligands and targets for molecular docking [26].

### Immunofluorescence

Primary Treg cells were collected and fixed in 4% formaldehyde, permeabilized with 0.5% Triton-X 100, blocked, and incubated with Stub1 and Foxp3 antibodies. Subsequently, cell nuclei were stained with Hoechst dye, and slides were imaged on a laser confocal microscope (Zeiss with LSM 900).

### Statistical analysis

Data were analyzed using GraphPad Prism 5 (GraphPad Software, San Diego, CA, USA) and represented as mean ± standard deviation (SD) of three independent experiments. Two-tailed Student’s t-test and one-way analysis of variance (ANOVA) were used as parametric tests. *P* < 0.05 (*), 0.01 (**), and 0.001(***) indicated a statistically significant difference.

## Results

### SAL treatment inhibits tumor growth, upregulates T cells and dendritic cells (DCs), and inhibits Tregs

**Fig. 1:**
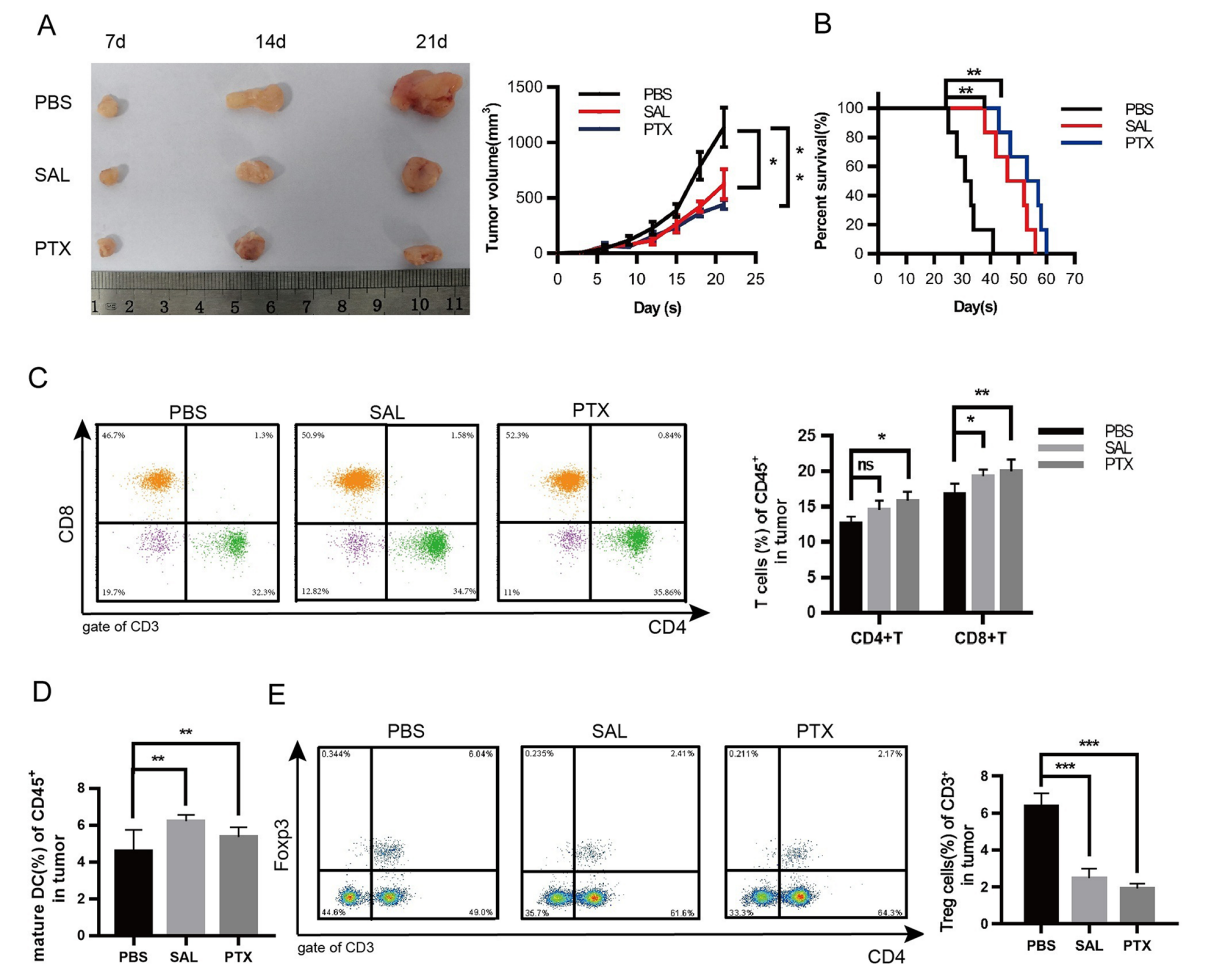
SAL reduces tumor progression and prolongs 3LL-bearing mice survival and decreases tumor-infiltrating immune cells. Tumor size **A** and survival rate **B** were recorded after PBS, SAL, or PTX treatment on tumor-bearing mice. Proportion of CD4^+^T, CD8^+^T **C**, DC **D**, and Treg cells **E** from the tumors was detected by flow cytometry. Three independent assays were performed; * *P* < 0.05, ** *P* < 0.01, *** *P* < 0.001

To assess the anti-tumor effect of SAL in lung cancer, SAL or PTX was used to treat the C57BL/6 mice bearing 3LL cells. At 12 days after SAL or PTX treatment, the tumor volume was decreased (Fig. 1A) compared to PBS treatment. SAL treatment prolonged the survival time of mice, which was similar to PTX treatment (Fig. 1B). These data showed the anti-tumor effect of SAL.

Furthermore, single-cell suspensions were prepared from the tumor tissues, and the cell types were analyzed by flow cytometry on day 21. The results showed that SAL treatment decreases the number of CD4^+^Foxp3^+^ T cells (Tregs) but increases the number of DCs and CD8^+^T cells infiltrating into tumor cells (Fig. 1C–E). The effect of SAL on tumor and tumor-infiltrating lymphocytes was similar to the effect of PTX (a positive control).

### SAL treatment downregulates Tregs by inhibiting Foxp3 expression

**Fig. 2:**
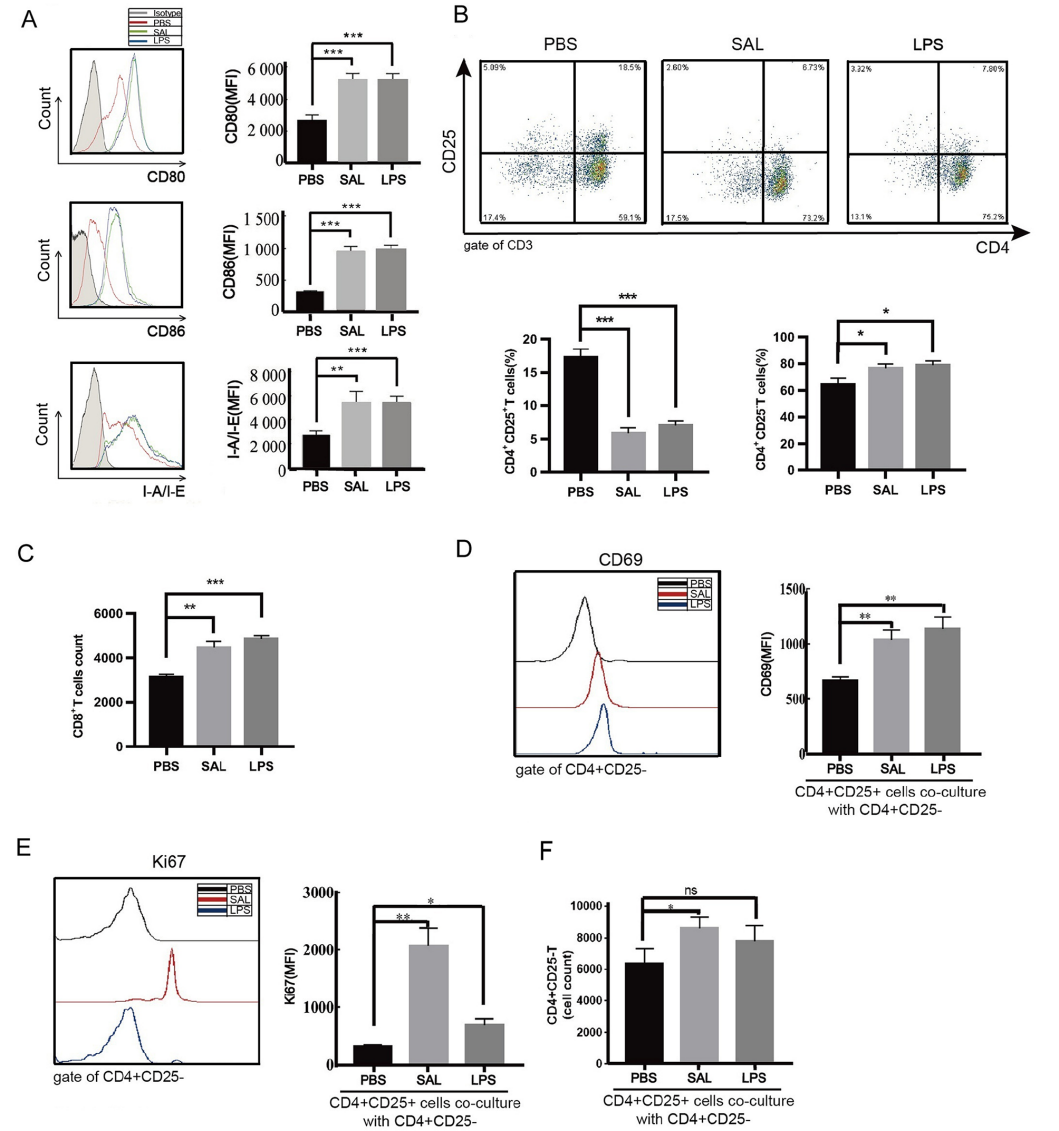
Effect of SAL on DCs and T cells in the in vitro experiments. **A** Expression levels of CD80, CD86, and I-A/I-E on isolated CD11c^+^MHC-II^+^ cells, treated with PBS, SAL, and LPS. **B** Percentage of CD4^+^CD25^−^and CD4^+^CD25^+^T cells from isolated CD4^+^T cells treated with PBS, SAL, and LPS. **C** Number of CD8^+^T cells treated with SAL, PBS, or LPS was counted. Expression of CD69 **D**, Ki67 **E** on CD4^+^CD25^−^T cells and the number of CD4^+^CD25^−^T cells **F** cocultured with SAL-, PBS-, or LPS-treated CD4^+^CD25^+^T cells. * P < 0.05, ** P < 0.01, ***P < 0.001

In vitro studies showed that SAL treatment promotes the maturation of DCs (Fig. 2A) and increases the number of CD4^+^CD25^−^T cells and CD8^+^T cells compared to PBS treatment, which is similar to the effect of LPS (a positive control) (Fig. 2B, C). However, the percentage of CD4^+^CD25^+^T cells is decreased in CD4^+^T cells (Fig. 2B). Moreover, CD4^+^CD25^+^T cells stimulated by SAL or LPS were cocultured with CD4^+^CD25^−^T cells in a ratio of 1:10 for 48 h. The expression of CD69 and Ki67 in CD4^+^CD25^−^T cells cocultured with SAL or LPS treated CD4^+^CD25^+^T cells was higher than that cocultured with PBS group (Fig. 2D-E). On the other hand, Tregs treated with SAL could not inhibit the proliferation of CD4^+^CD25^−^T cells compared to those treated with PBS (Fig. 2F), suggesting that SAL suppresses the inhibitory effect of Tregs.

**Fig. 3:**
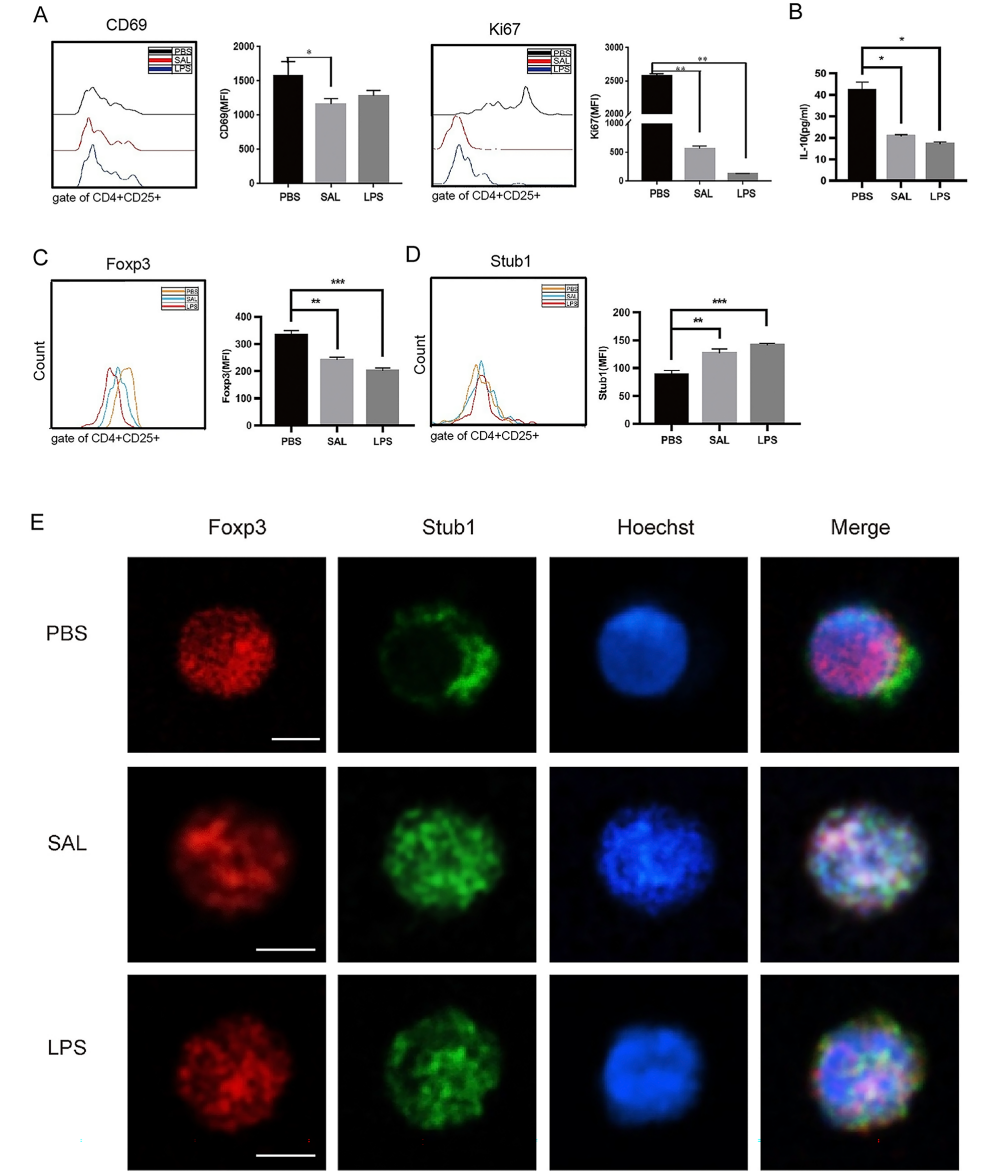
Effect of SAL on the expression and localization of Foxp3 and Stub1 in Tregs. **A** Expression of CD69 and Ki67 in Tregs stimulated by SAL, PBS, or LPS. **B** Concentration of IL-10 from SAL-, PBS-, or LPS-treated CD4^+^CD25^+^T cells. **C** Protein expression of Foxp3 in SAL-, PBS-, or LPS-treated Treg cells was analyzed by MFI. **D** MFI of Stub1 in SAL-, PBS-, or LPS-treated Tregs was measured by flow cytometry. **E** Stub1 enters into the nucleus and colocalizes with Foxp3 under SAL and LPS stimulation. Cell samples were harvested at the indicated time points and stained with APC-conjugated anti-Foxp3 antibody (Red) and anti-Stub1 antibody, followed by 488-labeled anti-rabbit antibody (Green) and nuclei stained with Hoechst (Blue). Scale bar is 3 μm. Representative findings are shown from at least three independent experiments. Three independent assays were performed; * P < 0.05, ** P < 0.01, *** P < 0.001

SAL treatment inhibited the expression of CD69 and Ki67 expression in Tregs (Fig. 3A). IL-10 secretion in SAL-treated Tregs was lower than those treated with PBS (Fig. 3B). The effect of SAL on Tregs was similar to that of LPS, which is a positive inhibitor on Tregs. Thus, it could be deduced that SAL activates immunity by inhibiting Tregs directly.

The X chromosome-linked, linage-specific transcription factor Foxp3 is a key transcription factor of Tregs. To analyze whether SAL treatment affects the expression of Foxp3 in Tregs, the expression of Foxp3 was detected in isolated Tregs post-SAL treatment. Subsequently, SAL treatment, but not PBS, decreased the protein expression of Foxp3 in Tregs (Fig. 3C). Thus, SAL treatment inhibits the protein expression of Foxp3 in Tregs.

### SAL treatment promotes the expression and intranuclear localization of Stub1 in Tregs

Because a stress-activated E3 ubiquitin ligase termed “Stub1” promoted the degradation of Foxp3 [23], the expression and localization of Stub1 in SAL-treated Treg were detected to find whether Stub1 could influence the expression of Foxp3. Stub1 expression was upregulated in SAL-treated mouse Treg cells, compared to PBS-treated mouse Treg (Fig. 3D). This phenomenon was similar to the previous finding of LPS effect in Treg cells [27].

Next, we characterized the colocalization of Foxp3 with Stub1 in Tregs. SAL treatment revealed that Stub1 is translocated into the nucleus and co-localized with Foxp3 (Fig. 3E), which was similar to LPS treatment. However, with PBS stimulation, Foxp3 is mainly localized in the nucleus, yet Stub1 in the cytoplasm (Fig. 3E). The translocation of Stub1 from the cytoplasm to the nucleus and the colocalization of Stub1 and Foxp3 in Treg after SAL treatment, suggested that SAL treated might promote Stub1-mediated Foxp3 degradation.

### SAL treatment regulates the expression of Hsp70

**Fig. 4:**
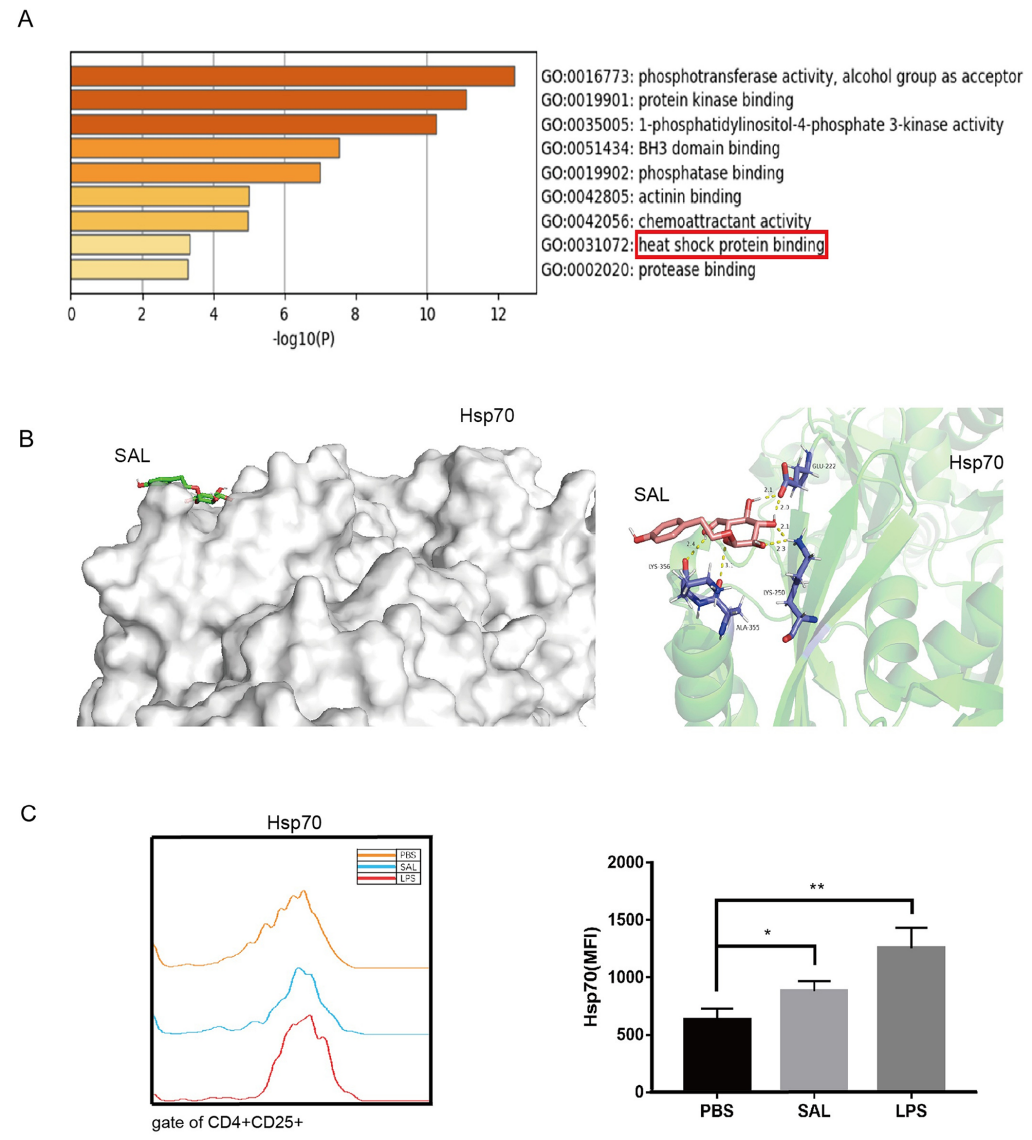
Hsp70 is a potential target of SAL. Prediction of HSP70 as the upstream target of SAL regulating Foxp3 by network pharmacology **A** and molecular docking technology **B**. **C** The expression of Hsp70 in SAL-, PBS-, or LPS-treated Tregs was measured by flow cytometry. * P < 0.05, ** P < 0.01

To analyze how SAL regulates the Stub1-mediated Foxp3 degradation, network pharmacology methods were used to analyze the target of SAL in NSCLC. GO enrichment analysis revealed that SAL exerted its anti-tumor effect by affecting the HSP binding sites (Fig. 4A). Hsp70 is known to recruit Stub1 to regulate the degradation of Foxp3 [23]. In addition, the docking results of SAL and Hsp70 molecules show that the SAL can enter and bind with the active pocket of Hsp70, and the binding of two proteins is high. The active sites of SAL and Hsp70 showed a compact binding pattern in the active pocket and interacted with amino acid residues LYS-356, ALA-355, LYS-250, and GLU-222 in Hsp70 via hydrogen bonds (Fig. 4B). The results of AutoDock docking showed that the binding energy of SAL and Hsp70 is -1.82 kcal/mol. When the binding energy is < 0 kcal/mol, the energy can be released in the docking process, and the docking can be completed without external intervention. These results showed that the binding energy of SAL with receptor protein Hsp70 is large, and the binding conformation is stable, thereby indicating a potential role of SAL in targeting Hsp70.

Furthermore, the effect of SAL on Hsp70 was detected. With SAL treatment on Tregs, the expression of Hsp70 increased, which was similar to LPS treatment. However, PBS could not upregulate the expression of Hsp70 in Tregs (Fig. 4C).

### Inhibiting Hsp70 reversed the inhibition of SAL on Foxp3

To analyze the role of Hsp70 on SAL-treated Tregs, the expression of Hsp70 was inhibited by HSP70-IN-1, and the expression and localization of Stub1 and Foxp3 in Treg with or without SAL treatment was detected.

**Fig. 5:**
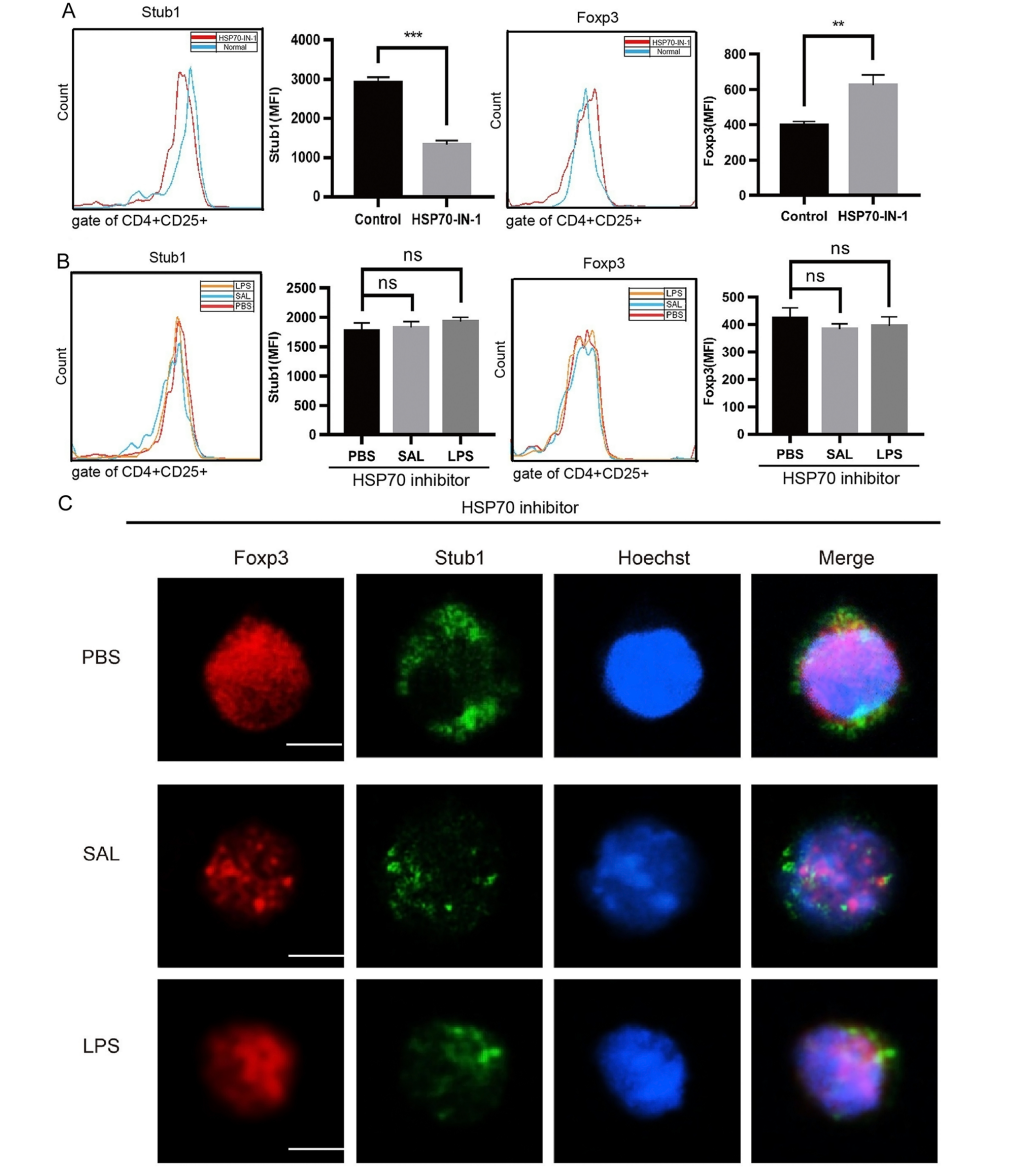
Inhibiting Hsp70 influences the expression of stub1 and Foxp3 in SAL-, PBS-, or LPS-treated Tregs or untreated Tregs. **A** MFI of Stub1 and Foxp3 in Tregs after 2.5 µM HSP70-inhibitor-1 (HSP70-IN-1) treatment for 48 h. **B** MFI of Stub1 and Foxp3 on SAL-, PBS-, or LPS-treated Tregs pre-treated with HSP70-IN-1. Three independent assays were performed: * P < 0.05, ** P < 0.01. **C** The same method was used to detect the intranuclear localization of Foxp3 and Stub1 in each group with HSP70 inhibitor. Scale bar is 3 μm

Inhibiting Hsp70 decreased the expression of Stub1 and increased the expression of Foxp3 (Fig. 5A). However, after inhibiting Hsp70, SAL or LPS treatment could not influence the expression of Foxp3 and Stub1 in Treg (Fig. 5B), and could not promote the translocation of Stub1 into nucleus (Fig. 5C). Taken together, SAL promotes the colocalozation of Stub1 and Foxp3 in Tregs by stimulating the expression of Hsp70.

## Discussion

In this study, we reported that SAL treated NSCLC by regulating the tumor microenvironment. SAL treatment increases the percentage of tumor-infiltrating DCs, CD4, and CD8^+^T cells, but decreases the percentage of Treg by inhibiting the expression of Foxp3 through Hsp70/Stub1/Foxp3 pathway. To the best of our knowledge, this is the first study showing that SAL promotes the expression of Hsp70 and Stub1 to downregulate the number and function of Tregs by degrading Foxp3 and further relieves the inhibition of effector T cells. Our previous findings have shown that SAL promotes the activation and expansion of DC and T cells, which might inhibit tumor growth [28]. Previous studies have mainly focused on the antitumor activity of SAL, such as inhibiting tumor cell migration, tumor cell proliferation, and oxidative stress response and activating apoptosis-related pathways [9, 29]. Although several studies have described SAL as an activator of monocytes and an antigen-presenting cell and activate the natural and Th1 immune response [12, 30], none of them reported the inhibitory effect of SAL on Tregs, which provides a novel idea for the application of SAL in antitumor therapy.

**Fig. 6:**
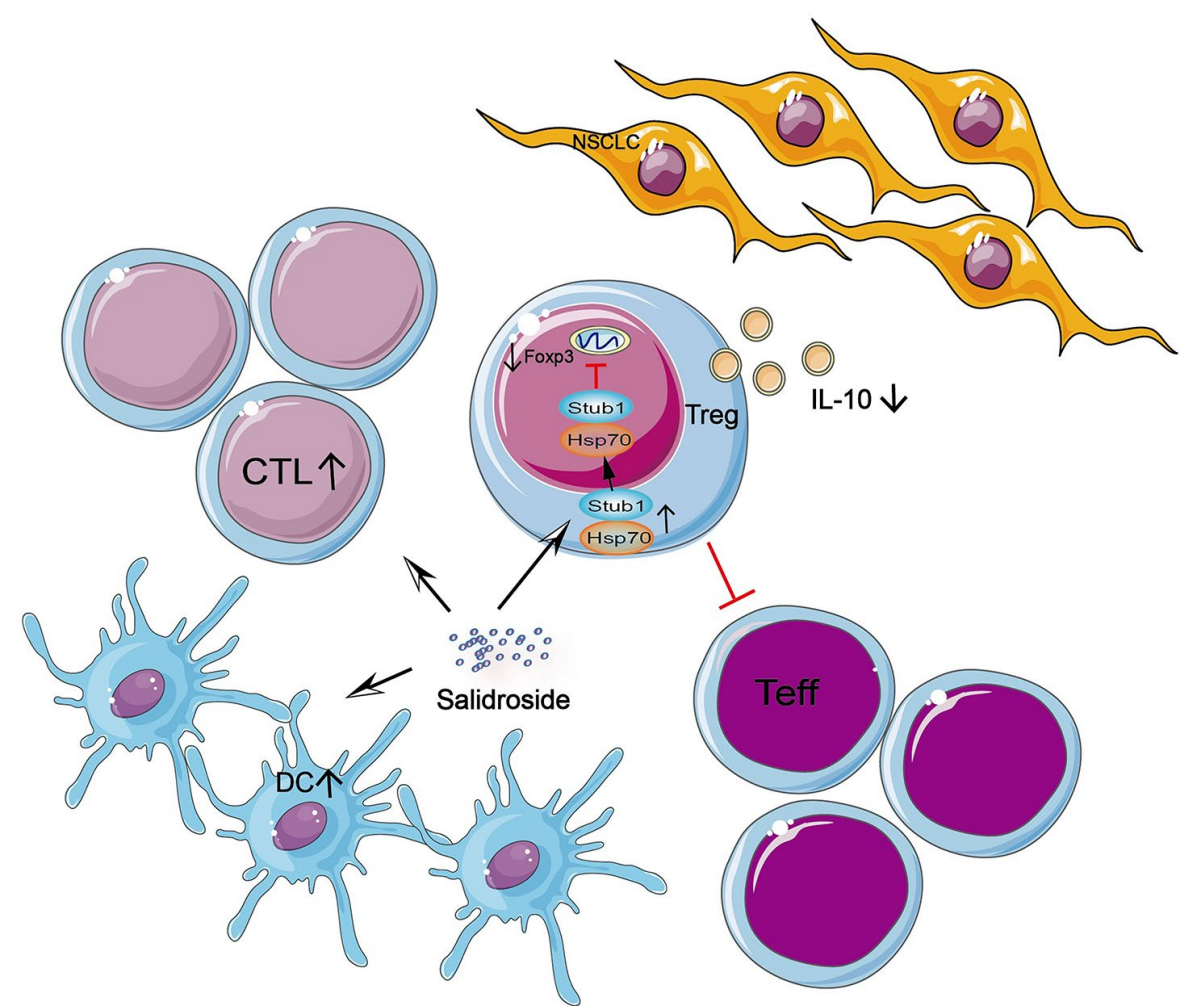
Schematic of the mechanism of salidroside-regulated tumor microenvironment by downregulating the expression of Foxp3 on regulatory T cells. SAL increases the expression of Hsp70 and Stub1 and transfers Hsp70 and Stub1 from outside into the nucleus, promotes the degradation of Foxp3, and inhibits the function of Tregs and tumor growth

Several studies have focused on how Foxp3 is induced in Treg cells, but the negative regulation of Foxp3 protein is yet to be elucidated. In this study, we explored how SAL downregulated the expression of Foxp3 in Tregs by regulating the post-translational modification of the protein, further promoting the degradation of Foxp3 in Treg. Previous studies have shown that the expression of E3 ligase Stub1 can ubiquitinate Foxp3 with the help of chaperone Hsp70, which leads to the degradation of the transcription factor in Treg [31, 32]. In the immune system, Hsp70 can activate the innate immune system [33, 34]. It also recruits Stub1 under inflammatory conditions to promote its transfer to the nucleus, further ubiquitinating and degrading Foxp3. Next, we determined that SAL treatment affected the expression of Hsp70. It might be SAL enters into the Treg through pinocytosis, and bind with the active region of Hsp70 to activate Hsp70. Inhibiting the expression of Hsp70 eliminated the effect of SAL on the expression of stub1 and Foxp3. Hence, SAL inhibits Tregs through Hsp70/Stub1/Foxp3 pathway (Fig. 6).

Consistent with previous reports [9, 11, 29], SAL directly inhibits the proliferation of tumor cells and induces tumor cell apoptosis, suggesting that SAL is an ideal drug for antitumor therapy. Moreover, SAL directly inhibits the proliferation of tumor cells [9, 11, 29] and regulates the activity and function of immune cells in the tumor microenvironment. In the mouse model of lung injury, SAL also regulated the secretion of inflammatory factors and the number of neutrophils and macrophages, further protecting LPS-induced lung injury [35]. These studies suggested that SAL has a regulatory effect on the immune response. It is possible that SAL can remission the tumor by regulating other immune cells. Whether SAL has other regulatory effects in tumor remains to be further studied. Nonetheless, the pharmacological mechanism of SAL on the disease needs to be studied further.

Above all, SAL is a traditional Chinese medicine extract that inhibits tumor growth by regulating the tumor immune microenvironment. Hsp70/Stub1/Foxp3 pathway promotes SAL-mediated Treg inhibition, thereby providing a new perspective for the antitumor effect and a novel strategy for the clinical treatment of SAL. It has been reported that Hsp70 and Hsp90 regulate TGF-β signaling by participating in Stub1-mediated ubiquitination and degradation of Smad3 [36]. Our previous study also found that SAL inhibited TGF-β secretion in Treg [37]. Whether SAL regulates TGF-β through Hsp70, and its possible mechanism, will be further investigated.

## Conclusions

This study describes the novel immunological mechanism of salidroside (SAL) on anti-tumor treatment. SAL regulates the tumor immune microenvironment by inhibiting the activation and function of Tregs and downregulating the expression of Foxp3 but increasing the number of CD8^+^T cells and effector CD4^+^T cells. SAL also stimulates the expression of Hsp70 and Stub1, further promoting the degradation of Foxp3. Thus, this study would contribute to assessing the immuno-pharmacological value of SAL in the treatment of non-small cell lung cancer.

## Electronic supplementary material

Below is the link to the electronic supplementary material.

Supplementary Material 1

## Data Availability

The datasets generated for this study are available upon request to the corresponding author.

## Abbreviations

DC: Dendritic cell
GO: Gene ontology
HSP: Heat shock protein
LPS: Lipopolysaccharide
NSCLC: Non-small cell lung cancer
PTX: Paclitaxel
SAL: Salidroside
SD: Standard deviation
Treg: Regulatory T cell
