# Supplementary material for: Salidroside regulates tumor microenvironment of non-small cell lung cancer via Hsp70/Stub1/Foxp3 pathway in Tregs
Source: BMC Cancer. 2023 Aug 1;23:717. doi: 10.1186/s12885-023-11036-5 (PMC10391887; doi:10.1186/s12885-023-11036-5)
Supplement: Supplementary file 1 — Supplementary Material 1 [file 12885_2023_11036_MOESM1_ESM.docx]

Supplemental Information


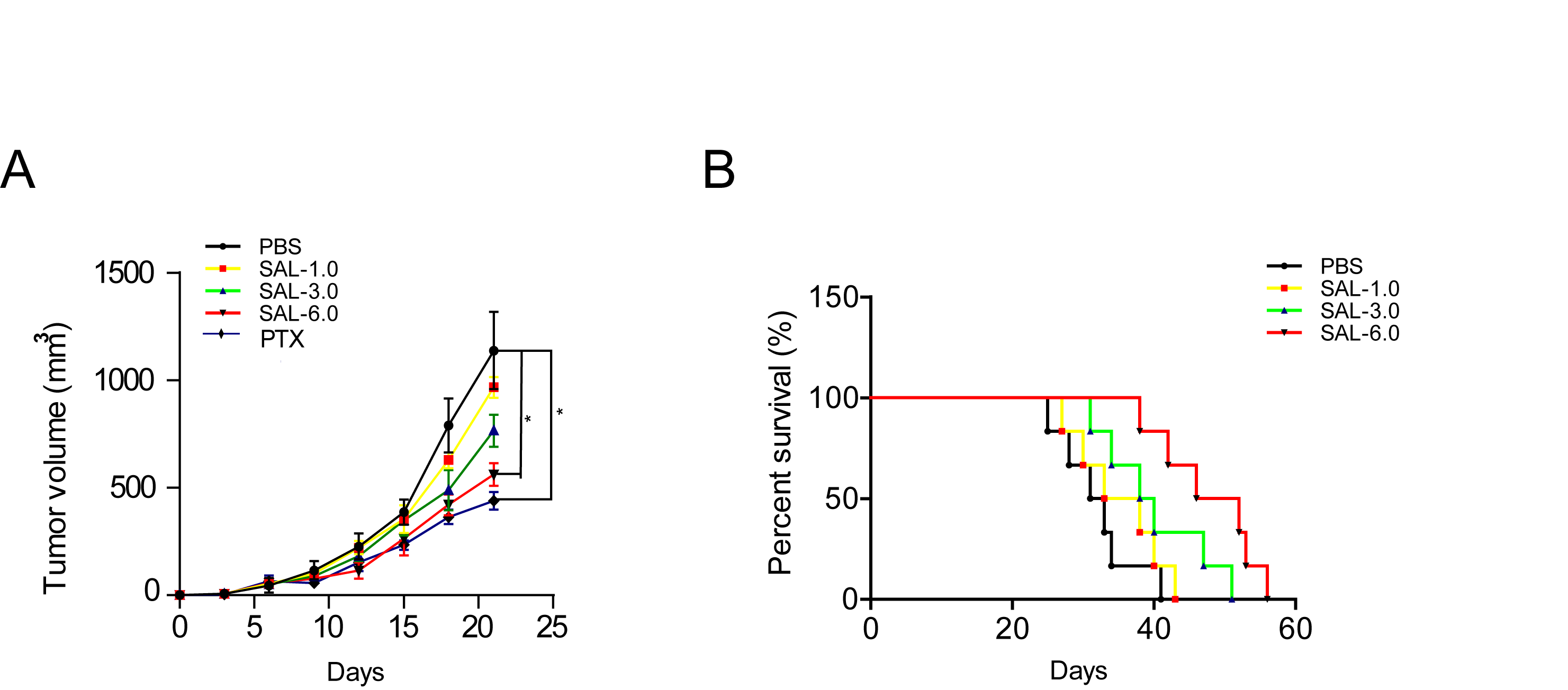


Supplementary Information Figure S1. Effects of different doses of SAL (1.0 mg/kg·d、3.0 mg/kg·d、6.0 mg/kg·d) on the tumor size of non-small cell lung cancer (**A**) and the survival rate of tumor-bearing mice (**B**). PTX (2.0 mg/kg·d) treatment as positive control, PBS as negative control. * *P*<0.05.


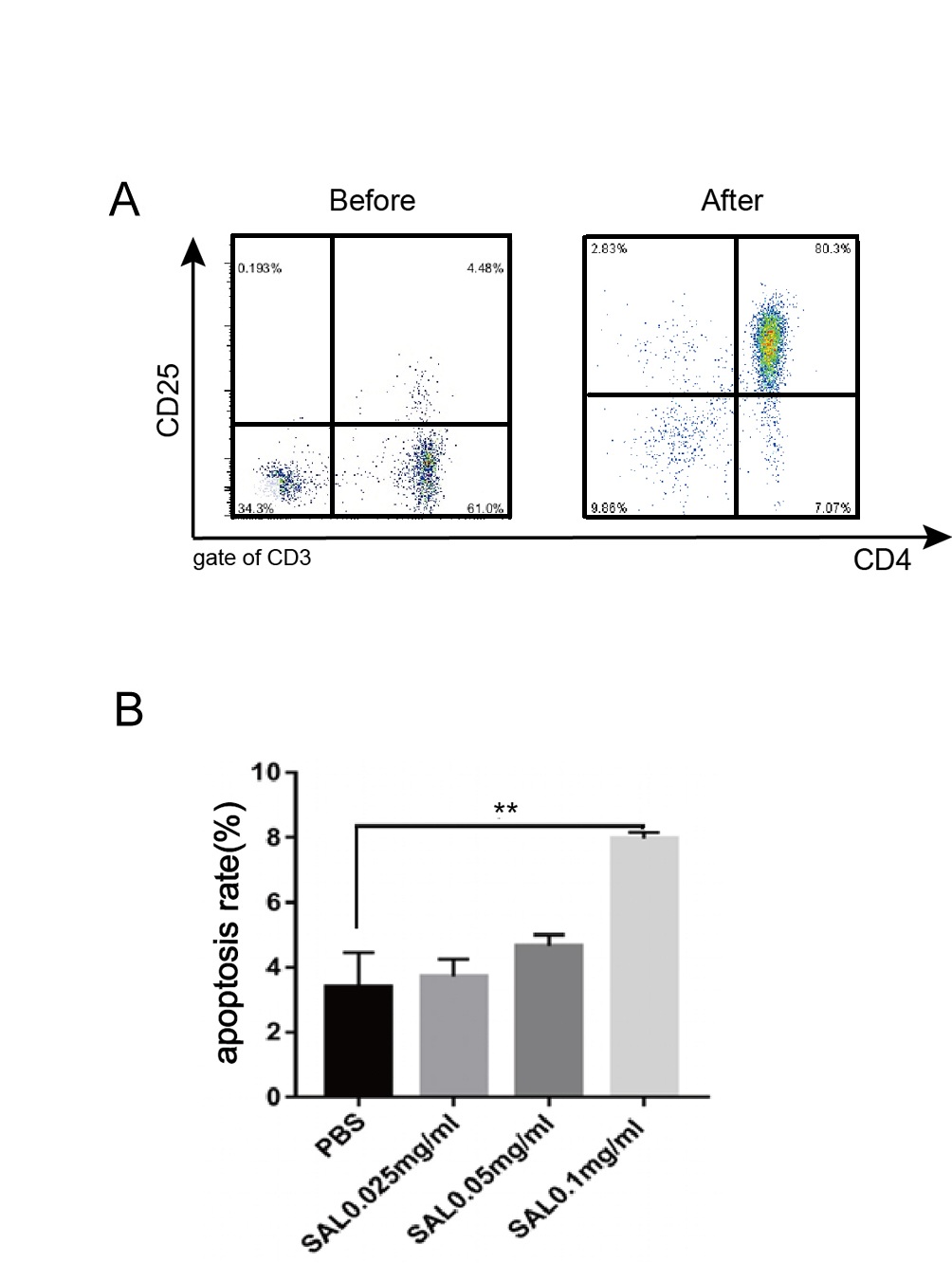


Supplementary Information Figure S2. Isolated Treg and the effect of SAL on Treg. (**A**) Purity of mouse spleen CD4^+^CD25^+^T cells sorted by magnetic beads. (**B**) The apoptosis rate of Treg stimulated by different concentrations of SAL was detected by flow cytometry. ** *P*<0.01.


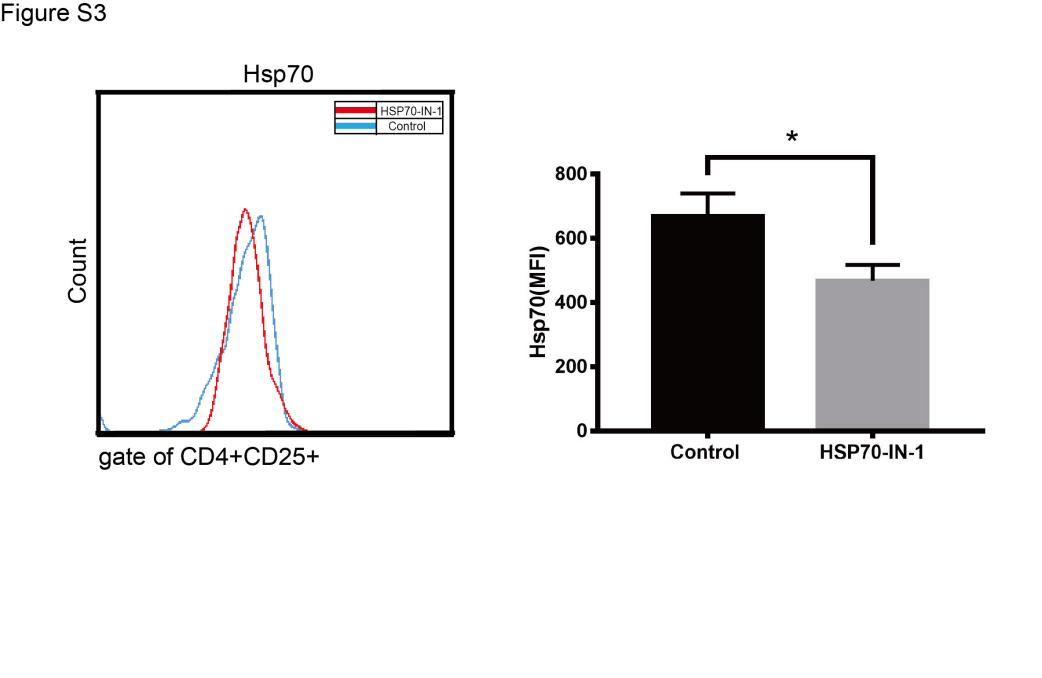


Supplementary Information Figure S3. The inhibitory effect of HSP70-IN-1 on Treg. The mean fluorescence value of Hsp70 in Treg which were treated with HSP70-IN-1 or not. * *P*<0.05.
